# Evaluating GPT and BERT models for protein–protein interaction identification in biomedical text

**DOI:** 10.1093/bioadv/vbae133

**Published:** 2024-09-11

**Authors:** Hasin Rehana, Nur Bengisu Çam, Mert Basmaci, Jie Zheng, Christianah Jemiyo, Yongqun He, Arzucan Özgür, Junguk Hur

**Affiliations:** Department of Computer Science, School of Electrical Engineering & Computer Science, University of North Dakota, Grand Forks, ND 58202, United States; Department of Biomedical Sciences, School of Medicine and Health Sciences, University of North Dakota, Grand Forks, ND 58202, United States; Department of Computer Engineering, Bogazici University, Istanbul 34342, Turkey; Department of Computer Engineering, Bogazici University, Istanbul 34342, Turkey; Unit for Laboratory Animal Medicine, University of Michigan, Ann Arbor, MI 48109, United States; Department of Biomedical Sciences, School of Medicine and Health Sciences, University of North Dakota, Grand Forks, ND 58202, United States; Unit for Laboratory Animal Medicine, University of Michigan, Ann Arbor, MI 48109, United States; Center for Computational Medicine and Bioinformatics, University of Michigan, Ann Arbor, MI 48109, United States; Department of Computer Engineering, Bogazici University, Istanbul 34342, Turkey; Department of Biomedical Sciences, School of Medicine and Health Sciences, University of North Dakota, Grand Forks, ND 58202, United States

## Abstract

**Motivation:**

Detecting protein–protein interactions (PPIs) is crucial for understanding genetic mechanisms, disease pathogenesis, and drug design. As biomedical literature continues to grow rapidly, there is an increasing need for automated and accurate extraction of these interactions to facilitate scientific discovery. Pretrained language models, such as generative pretrained transformers and bidirectional encoder representations from transformers, have shown promising results in natural language processing tasks.

**Results:**

We evaluated the performance of PPI identification using multiple transformer-based models across three manually curated gold-standard corpora: Learning Language in Logic with 164 interactions in 77 sentences, Human Protein Reference Database with 163 interactions in 145 sentences, and Interaction Extraction Performance Assessment with 335 interactions in 486 sentences. Models based on bidirectional encoder representations achieved the best overall performance, with BioBERT achieving the highest recall of 91.95% and F1 score of 86.84% on the Learning Language in Logic dataset. Despite not being explicitly trained for biomedical texts, GPT-4 showed commendable performance, comparable to the bidirectional encoder models. Specifically, GPT-4 achieved the highest precision of 88.37%, a recall of 85.14%, and an F1 score of 86.49% on the same dataset. These results suggest that GPT-4 can effectively detect protein interactions from text, offering valuable applications in mining biomedical literature.

**Availability and implementation:**

The source code and datasets used in this study are available at https://github.com/hurlab/PPI-GPT-BERT.

## 1 Introduction

Protein-protein interactions (PPIs) are essential for numerous biological functions, especially DNA replication and transcription, signaling pathways, cell metabolism, and converting genotype to phenotype. Gaining insight into these interactions enhances understanding of the biological processes, pathways, and networks underlying healthy and diseased states. Various public PPI databases exist (Alonso-López *et al.* 2016, Szklarczyk *et al.* 2019, Oughtred *et al.* 2021), including PPI data collected from low-to-mid throughput experiments such as yeast-two-hybrid or immunoprecipitation pull-down or high-throughput screening assays. However, these resources are incomplete and do not cover all potential PPIs. Supplementary Figure S1 illustrates PPI information extraction from biomedical literature. Due to the rapid growth of scientific literature, manual extraction of PPIs has become increasingly challenging, necessitating automated text mining approaches that do not require human participation.

Natural language processing (NLP) is a focal area in computer science, increasingly applied in various domains, including biomedical research, which has experienced massive growth in recent years. Relation extraction, a widely used NLP method, aims to identify relationships between two or more entities in biomedical text, supporting the automatic analysis of documents in this domain (Li *et al.* 2021). Advances in deep learning (DL), such as convolutional neural networks (CNNs) (Peng and Lu 2017, Choi 2018) and recurrent neural networks (RNNs) (Hsieh *et al.* 2017, Ahmed *et al.* 2019), as well as in NLP, have enabled success in biomedical text mining to discover interactions between protein entities. The field has also seen advancements through pretraining large neural language models, leading to substantial improvements in various NLP problems (Gao *et al.* 2023). Following the seminal work “Attention Is All You Need” (Vaswani *et al.* 2017), transformer architectures have set new benchmarks in various NLP tasks, including relation extraction in the biomedical domain (Gu *et al.* 2021).

After the development of transformer architecture (Vaswani *et al.* 2017), transformer-based models like bidirectional encoder representation transformer (BERT) (Devlin *et al.* 2019), a type of masked language model, emerged. These models, known as large language models (LLMs), focus on understanding language and semantics. LLMs are pretrained on vast amounts of data and can be fine-tuned for various tasks. Recent studies suggest that LLMs excel at context zero-shot and few-shot learning (Lee *et al.* 2020), analyzing, producing, and comprehending human languages. LLMs’ massive data processing capabilities can be used to identify connections and trends among textual elements. Recent studies (Warikoo *et al.* 2021, Park *et al.* 2022, Roy and Mercer 2023) used domain-specific pretrained BERT models on gold-standard PPI datasets, including Learning Language in Logic (LLL) (Nédellec 2005), a subset of Human Protein Reference Database (HPRD50) (Fundel *et al.* 2007), Interaction Extraction Performance Assessment (IEPA) (Ding *et al.* 2022), AIMED (Bunescu *et al.* 2005), and Bio Information Extraction Resource (BioInfer) (Pyysalo *et al.* 2007), to capture relations between protein entities present in a given text. In another research (Roy and Mercer 2023), classification performance was improved using tree transformers and a graph neural network. Furthermore, researchers developed a lexically-aware Transformer-based Bidirectional Encoder Representation (Warikoo *et al.* 2021), advancing our understanding of bio-entity relation extraction by introducing a versatile model that investigates both local and global contexts for sentence-level classification tasks.

Another type of LLM is autoregressive language models, including generative pretrained transformer (GPT), an advanced artificial intelligence (AI) language model that generates human-like text by acquiring linguistic patterns and structures. GPT (Radford *et al.* 2018) is a series of language models, developed by OpenAI in 2018, based on transformer architecture (Vaswani *et al.* 2017). The transformer model consists of an encoder that generates latent representations and a decoder that produces output sequences using multi-head attention, which prioritizes data over inductive biases, facilitating large-scale pretraining and parallelization. The self-attention mechanism enables neural networks to determine the importance of input elements, making it ideal for language translation, text classification, and generation. GPT-1, the first version of GPT, with 117 million parameters, was trained using a large corpus of text data, including Wikipedia (https://en.wikipedia.org/), Common Crawl (https://commoncrawl.org/the-data/), and OpenWebText (https://skylion007.github.io/OpenWebTextCorpus/). GPT-2 (Radford *et al.* 2019) significantly improved over its predecessor with roughly ten times bigger 1.5 billion parameters. Training on a larger corpus of text data, including web pages and books, could generate more coherent and convincing language responses. GPT-3 (Brown *et al.* 2020) was trained with 175 billion parameters, including an enormous corpus of text data, web pages, books, and academic articles. GPT-3 has demonstrated outstanding performance in various NLP tasks, such as language translation, chatbot development, and content generation.

On 30 November 2022, OpenAI released ChatGPT, a natural and engaging conversation tool capable of producing contextually relevant responses based on text data. ChatGPT was fine-tuned on the GPT-3.5 series. On 14 March 2023, OpenAI introduced its most advanced and cutting-edge system, GPT-4, which has surpassed its predecessors by producing more dependable outcomes. GPT-4 (OpenAI 2023) is now a multi-modal system capable of processing not only text but also images and voice inputs, enhancing its ability to engage in various creative and technical writing tasks, such as songwriting, screenplay creation, and imitating user writing styles (Cheng *et al.* 2023). The advancements in GPT models, from GPT-3 to GPT-4, showcase the rapid progress in NLP, opening up a wide range of applications (Baktash and Dawodi 2023, Microsoft Research AI4Science and Microsoft Azure Quantum 2023).

Several studies (Katz *et al.* 2024, Nori *et al.* 2023) have evaluated the performance of GPT models for problem-solving on various standardized tests. It has been shown that they can achieve performance comparable to or even better than humans (Webb *et al.* 2023). They could pass high-level professional standardized tests such as the Bar test (Kojima *et al.* 2022), the Chinese Medical Practitioners examination (Wang *et al.* 2023), and the Japanese National Nurse examination (Taira *et al.* 2023). Another study (Gilson *et al.* 2023) evaluates that ChatGPT achieves the equivalent of a passing score for a third-year medical student in the United States Medical Licensing Examination. A group of researchers explored the performance of advanced LLMs, including ChatGPT, GPT-4, and Google Bard, in mastering complex neurosurgery examination material and found that GPT-4 achieved a score of 82.6%, outperforming ChatGPT and Google Bard. A recent study assessed the efficacy of GPT for specific standardized admissions tests in the United Kingdom. These findings revealed that GPTs demonstrate greater proficiency in tasks requiring language comprehension and processing and yet exhibit limitations in applications involving scientific and mathematical knowledge (Giannos and Delardas 2023). To the best of our knowledge, no study has evaluated the effectiveness of GPT models, specifically in extracting PPIs from biomedical texts. Here, we present a thorough evaluation of the PPI identification performance of the GPT-based models and compare these with the state-of-the-art BERT-based NLP models.

## 2 Methods

### 2.1 Language models

We evaluated two autoregressive language models (GPT-3.5 and GPT-4), each with six variations, and three masked language models (BioBERT, PubMedBERT, and SciBERT). The overall methodology of our research is illustrated in Supplementary Fig. S2.

#### 2.1.1 Autoregressive language models

The GPT architecture includes layers with self-attention mechanisms, fully connected layers, and layer normalization, reducing computational time and preventing overfitting during training (Radford *et al.* 2018). In our study, we have used the version released on 13 June 2023, for GPT-3.5 and GPT-4 (gpt-3.5–0613 and gpt-4–0613, respectively). The architecture and number of parameters for GPT models used in our experiment are summarized in Table 1, including the GPT-3.5 and GPT-4 models, which were included in the current study.

**Table 1.** Specifications of GPT and BERT-based models.

Model Type	Model name	Year released	Architecture	Number of parameters	Context window	Accessed through
***Autoregressive Language Models***	GPT-3.5	2022	A combination of three models: code-davinci-002, text-davinci-002, and text-davinci-003.	1.3 billion, 6 billion, and 175 billion	4096 tokens	OpenAI API
	GPT-4	2023	Fine-tuned using reinforcement learning from human feedback.	Supposedly 100 trillion	8192 tokens	OpenAI API
***Masked Language Models***	BioBERT	2019	Encoder architecture of transformer with 12 layers and hidden size of 768	108 million	512 tokens	Hugging Face (dmis-lab/biobert-v1.1)
	SciBERT	2019	Encoder architecture of transformer with 12 layers and hidden size of 768	108 million	512 tokens	Hugging Face (allenai/scibert_scivocab_uncased)
	PubMedBERT	2020	Encoder architecture of transformer with 12 layers and hidden size of 768	108 million	512 tokens	Hugging Face (microsoft/BiomedNLP-PubMedBERT-base-uncased-abstract)

#### 2.1.2 Masked language models

Three different BERT-based models were included in the current study (Table 1). There are various BERT-based models that are either trained or fine-tuned on biomedical corpora. We have selected the BioBERT (Lee *et al.* 2020), SciBERT (Beltagy *et al.* 2019), and PubMedBERT (Gu *et al.* 2021) models, which are commonly used for biomedical text processing. BioBERT (Lee *et al.* 2020) is a BERT model pretrained on PubMed abstracts and PubMed Central (PMC) full-text articles for different NLP tasks to measure performance. The initial version, BioBERT v1.0, used >200K abstracts and >270K PMC articles. An expanded version of BioBERT v1.1 was fine-tuned using > 1M PubMed abstracts and was included in the current study. The model was accessed from the following Hugging Face repository: dmis-lab/biobert-v1.1. SciBERT (Beltagy *et al.* 2019) is a BERT model pretrained on random Semantic Scholar articles (Ammar *et al.* 2018). While pretraining with the articles, the entire text was used. The researchers created SCIVOCAB from scientific articles of the same size as BASEVOCAB, the BERT-based model’s vocabulary. Uncased SCIVOCAB was used in the current study. The model was accessed from the following Hugging Face repository: allenai/scibert_scivocab_uncased. PubMedBERT (Gu *et al.* 2021) is a BERT model trained explicitly on BLURB (Biomedical Language Understanding & Reasoning Benchmark). In this study, we used PubMedBERT, which was trained only on abstracts. The model was accessed from the following Hugging Face repository: microsoft/BiomedNLP-PubMedBERT-base-uncased-abstract.

### 2.2 Dataset

We used three widely used gold-standard datasets for PPI extraction: LLL (Nédellec 2005), IEPA (Ding *et al.* 2022), and HPRD50 (Fundel *et al.* 2007). These datasets offer a unique perspective and challenge in the field of biomedical NLP research, particularly in the area of PPI extraction. Originally released as part of the LLL shared task challenge in 2005, the LLL dataset is focused on extracting protein/gene interactions from biological abstracts related to *Bacillus subtilis*. The dataset contains 77 sentences with 164 manually annotated positive protein pairs (i.e. interacting protein pairs), also referred to as positive pairs in this study, and 166 negative pairs (i.e. noninteracting protein pairs). The IEPA dataset comprises nearly 300 abstracts from MEDLINE, utilizing specific biochemical noun queries. It includes 486 sentences with 335 positive pairs and 482 negative pairs. The dataset is a valuable resource for assessing the performance of PPI extraction models, given its diverse range of abstracts and detailed annotation of protein interactions. The HPRD50 dataset, a subset of HPRD, consists of 50 randomly selected abstracts. The dataset is annotated for various interactions, including direct physical interactions, regulatory relations, and modifications such as phosphorylation. It contains 145 sentences, with 163 positive and 270 negative pairs (Table 2).

**Table 2.** Number of positive and negative pairs in the sentences of the three datasets.

Dataset	Sentences	Positive pairs	Negative pairs	Total pairs	Ratio of positive and negative pairs
LLL	77	164	166	330	1:1.01
IEPA	486	335	482	817	1:1.44
HPRD50	145	163	270	433	1:1.65

Among these three datasets, LLL is the most focused and the least complex due to its size and high interaction density. It includes roughly equal numbers of positive and negative pairs. In contrast, IEPA and HPRD50 have their own intricacies in terms of directness and explicitness of interactions with more negative pairs than positive ones. IEPA has a significant portion of indirect interactions and a high percentage of explicit interactions, suggesting detailed but complex annotation.

We preprocessed the sentences in these gold-standard datasets. For the GPT models, protein dictionaries for each dataset were compiled by listing all the unique protein mentions. We also applied preprocessing steps to ensure capturing all entities by removing punctuation marks, digit-only strings, and blank spaces and converting all the letters into lowercase, resulting in a normalized protein dictionary. Both the original and normalized versions of dictionaries are utilized as auxiliary resources in our experiments with GPT-based models. For the BERT-based models, similar to the prior work (Erkan *et al.* 2007), we replaced the entity pair names with the *PROTEIN1* and *PROTEIN2* keywords. Other than the pair, the protein names in the sentence were substituted with the *PROTEIN* keyword. Although GPT models are not masked language models, we have evaluated them using both the original sentences and those where protein names were substituted with the PROTEIN keyword, similar to the approach used for BERT-based models.

We split each gold-standard dataset into ten folds using a document-level folding strategy, as previously described (Airola *et al.* 2008). The same ten folds were utilized for both GPT and BERT-based models. In the *PROTEIN* masked settings, sentences were repeated with the placeholders *PROTEIN1*, *PROTEIN2*, and *PROTEIN* positioned in various locations to represent different protein pairs within the same sentence. This procedure, however, resulted in minor variations of the same sentence, posing a potential challenge for GPT model interpretation. To mitigate this issue, we also implemented an N-fold partitioning strategy in our dataset processing. This approach was designed to ensure that no individual partition contained duplicated sentences with varied placements of protein placeholders, thereby reducing the likelihood of model confusion in distinguishing between these slight sentence variations.

### 2.3 Prompt engineering for GPT models

To extract PPIs from input sentences, we leveraged OpenAI’s application programming interface (API) access for the GPT models. We carefully designed the prompts to generate well-structured and stable interactions with minimal post-processing steps. We initiated this process by developing a foundational prompt consisting of seven sections. These seven sections are listed in Table 3.

**Table 3.** Structured breakdown of a complete prompt for step-by-step prompt engineering.

Section number	Purpose	Details
1	Primary instruction about extracting PPI	Extract every pair of PPI from the sentences provided as input, analyzing each sentence individually.
2	Term equivalence	For this task, “Proteins,” and “Genes” are synonymous.
3	Handling multiple PPI pairs from a single sentence	If a sentence contains multiple PPI pairs, list each pair on a distinct row.
4	Output format	Please, format your results in CSV (comma-separated values) format with the following four columns: “Sentence ID,” “Protein 1,” “Protein 2,” and “Interaction Type.” Ensure that no columns are left blank.
5	Output format column specification	Output Column Specifications: “Sentence ID”: The unique identifier for each sentence. “Protein 1” and “Protein 2”: The entities in the sentence, representing the proteins or genes. “Interaction Type”: The type of interaction identified between the protein entities (e.g., “binds to,” “inhibits”).
6	End-of-process indication	If all sentences have been processed successfully, the last row should only contain the word “Done.”
7	Input sentences	Each input line contains a “Sentence ID” and corresponding “Sentence” that is needed to be analyzed for finding PPI.Here are the sentences that you need to process:

In our approach to prompt engineering, we emphasized creating a diverse array of prompt variations for each section to pinpoint the most effective formats. The core of this approach was in Section 1, which is critical as it contains the primary instructions for the PPI identification task. We developed a comprehensive set of 128 variations for this section. For the subsequent sections, we generated fewer variations: 54 for Section 2, 96 for Section 3, 72 each for Sections 4 and 5, 24 for Section 6, and 12 for Section 7. We incorporated the variations in the foundational prompt while maintaining the other sections constant during section-wise testing. Then, we performed a section-wise evaluation to find the best variation of prompt from each section. The F1 score was used as the performance metric to select an optimal prompt for further analysis. The evaluation process was done sequentially, examining each section individually to select the most effective prompt variation before progressing to the next. This systematic approach ensured that each section’s best variation was carried forward into subsequent tests.

After identifying the top prompt from each section, we conducted a secondary analysis of these seven prompts. This step was crucial to mitigate any potential temporal biases that might have arisen from varying times of API access. The detailed algorithm for this prompt engineering process is depicted in Supplementary Fig. S3. Given the extensive nature of prompt engineering and the associated costs, we opted to utilize GPT-3.5 for prompt engineering instead of the more expensive GPT-4. To expand the scope of our study, we introduced two additional prompt variations that incorporate dictionaries of the original or normalized protein mentions. This strategy was chosen to assess the impact of additional domain-specific information on GPT models, as they were not explicitly trained for biomedical analysis or PPI identification. Further details regarding the composition and structure of these protein dictionaries can be found in Supplementary Table S1.

For protein-masked inputs, the base prompt was adapted to suit the altered input-output pattern. This adaptation involved experimenting with variations in the different sections of the prompts, ultimately selecting the variant with the highest F1 score for our analysis. In all scenarios, while using GPT models, we provided the sentence IDs and sentences as input, along with a prompt. Due to the context window limitations of GPT models, some of the larger folds were split into two (HPRD50) or three (IEPA) parts when the total number of tokens in input and estimated output exceeded the maximum token limit of the models. However, we combined the output parts from the same folds together before calculating performance scores. For all the model settings, we conducted ten independent runs and calculated their averages to mitigate any biases. To avoid the potential bias of accessing the GPT models at different times, parallel processing of all folds was also implemented. This involved generating a randomized list of all possible combinations of dataset folds, models, and prompt types for each run, thereby ensuring a balanced and unbiased experimental setup.

### 2.4 Temperature parameter optimization

OpenAI’s API allows the modulation of the “temperature” parameter in GPTs, which determines how greedy or creative the generative model is. The parameter ranges between 0 (the most precise) and 2 (the most creative) for GPT-3.5 and GPT-4. In our initial consideration for exploring the impact of temperature on PPI identification using the OpenAI API, we set the range of temperature settings from a minimum of 0 to a maximum of 2. However, we found that as the temperature increased above 1, the failure rate in generating output in the required format also increased along with performance degradation. Consequently, we revised our approach and limited the range from 0 to 1 for further analysis, taking into account the longer processing times associated with higher temperature settings.

### 2.5 Performance evaluation

In our study, BERT-based models were fine-tuned in a 10-fold cross-validation setting. This strategy used document-level fold splitting, which ensured the sentences from one document were used only either in the training or testing set to avoid overfitting (Mooney and Bunescu 2005). All fine-tuning experiments were conducted on the Tesla A100. We experimented with 1e−5, 2e−5, and 5e−5 as learning rate, 0.1, 0.01, and 0.001 as weight decay, and 4, 6, 8, and 32 as batch size. To provide fixed length input to the models, 128 and 256 were used as the maximum context length. When each example in each dataset was tokenized with the BERT tokenizers, the token length was <200, with one exception. If the token length of the sentence is greater than the length of the context window, the sentence is truncated. This led to a loss of information. Since we wanted to include the entire sentence in the model, we used 256 as the context length, which resulted in better performance compared to 128. On the other hand, we did not want to create input representations that consist mainly of filling information. Therefore, we did not choose 512 as the context length. Notably, fine-tuning with a maximum context length of 256 is faster than 512. We selected the best hyperparameters based on the average F1 score for models on all datasets’ validation splits. The hyperparameter search space and the best hyperparameters are shown in Table 4.

**Table 4.** Hyperparameter selection for K-Fold cross-validation on BERT models.

Hyperparameter	Search space	Best value
Optimizer	–	AdamW
Weight decay	0.1, 0.01, 0.001	0.01
Learning rate	1e−5, 2e−5, 5e−5	2e−5
Batch size	4, 6, 8, 32	4
Epochs per fold	1–10	8
Max sentence length	128, 256	256

## 3 Results

### 3.1 Prompt engineering for GPT models

The performance of all the variations of Prompts from each section is visualized in Supplementary Figs S4–S10. The most effective prompt from each of the seven sections, identified through extensive testing, is presented in Supplementary Table S2. Among these seven best prompts from each section, Prompt 60 from Section 3 (P60_S3) performed best in terms of F1 score. So, we selected P60_S3 as the base prompt and adjusted that to get the other settings. The final prompts of six different types of settings (base prompt, base prompt with protein dictionary, base prompt with normalized protein dictionary, *PROTEIN* masked prompt, *PROTEIN* masked prompt with no repeated sentence in same prompt, and *PROTEIN* masked prompt with one input sentence at a time) are detailed in Supplementary Table S3. However, the protein dictionaries mentioned in the tables under “with protein dictionary” and “with normalized protein dictionary” apply to the LLL dataset. For the other datasets, the specified dictionary is adapted accordingly.

### 3.2 Temperature parameter optimization

**Figure 1.**
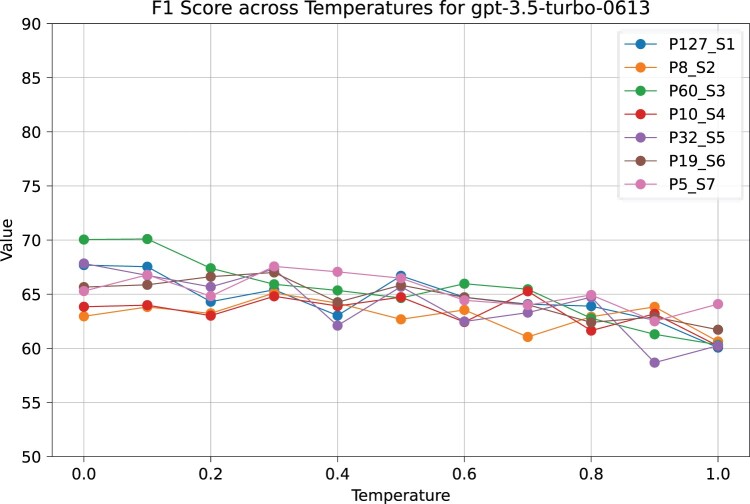
Performance evaluation of temperature parameter of GPT-3.5–0613 for best prompts from each section. PX_SY means Prompt number X from Section Y.

A temperature of 0.0 demonstrated the highest overall performance of GPT-3.5–0613 with the most consistent output format. Figure 1 depicts the F1 score variation of GPT-3.5 over a temperature range from 0 to 1.0. Supplementary Figures S11 and S12 represent the corresponding Precision and Recall variation.

**Figure 2.**
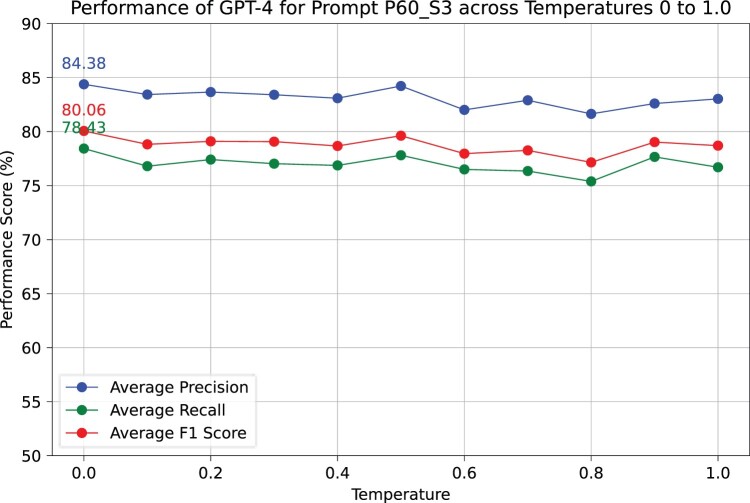
Performance evaluation of temperature parameter of GPT-4–0613 for Prompt60 of Section 3.

For GPT-4–0613, we have only explored the final base prompt due to the high expense of GPT-4. In Fig. 2, the precision, recall, and F1 scores were the highest, with a temperature of 0.0, although the differences were minor. However, at higher temperatures, the randomness of the output increases, causing output’s structure to become inconsistent and necessitating additional post-processing. Therefore, in our current study, a temperature setting of 0.0 was chosen for both GPT-3.5 and GPT-4 to have robust output.

### 3.3 PPI identification performance

The experimental analysis centers on the comparative performance of autoregressive language models, specifically GPT-3.5 and GPT-4 variants, against masked language models like BioBERT, PubMedBERT, and SciBERT in a PPI prediction task.

**Figure 3.**
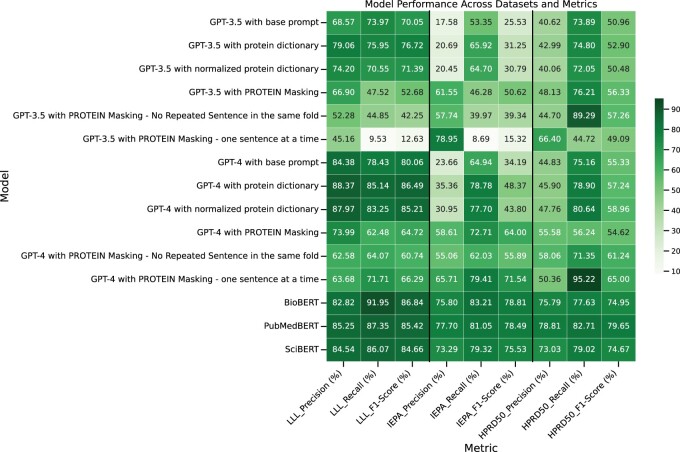
Evaluation result of PPI identification task on the LLL, IEPA, and HPRD50 datasets for BERT and GPT-based models.

The heatmap in Fig. 3 presents a comparative analysis of performance on the LLL, IEPA, and HPRD50 datasets. For LLL dataset, GPT-4, especially when enhanced with a Protein dictionary, exhibits remarkable strengths. It stands out with its precision rate of 88.37%, which notably exceeds that of the BERT-based models. This high precision indicates GPT-4’s ability to identify relevant information accurately, a crucial aspect in data processing and analysis. While its recall and F1 score are slightly lower than the BioBERT model, trailing by a mere 6.81% and 0.35%, respectively, this small margin highlights GPT-4’s balanced proficiency in both precision and F1 score. Furthermore, GPT-4’s recall rate, though lower than PubMedBERT’s, is still impressive at 85.14%. Supplementary Figure S13 presents a comparative visualization of the performance of BERT and GPT-based models in PPI identification on the LLL dataset. Complete evaluation scores for the LLL dataset can be found in Supplementary Table S4.

For the IEPA dataset, again, BERT-based models outperform GPT-based models on recall and F1 metrics. Supplementary Figure S14 illustrates a comparative performance on the IEPA dataset. Among all settings of GPT-based models, GPT3.5 with *PROTEIN* masking (one sentence at a time) has the highest precision (78.95%), and GPT-4 with *PROTEIN* masking (one sentence at a time) has the highest recall (79.41%) and F1 score (71.54%) for IEPA dataset. The use of domain-specific dictionaries and normalization improves the performance of the models, suggesting that incorporating domain knowledge is beneficial. However, despite these improvements, it is the masked language models that demonstrate superior overall efficacy, with BioBERT model’s 83.21% recall and 78.81% F1 score and PubMedBERT model’s 77.70% precision. The complete evaluation scores on the IEPA dataset for these models are listed in Supplementary Table S5.

For the HPRD50 dataset, GPT-4 with *PROTEIN* masking (one sentence at a time) impressively achieves the best recall of 95.22%. However, both GPT-3.5 and GPT-4 lag behind BERT-based models in terms of precision and F1 score. Among all the BERT-based models, PubMedBERT achieved the highest performance metrics with a precision of 78.81%, a recall of 82.71%, and an F1 score of 79.65% for the HPRD50 dataset. Thus, PubMedBERT has the highest overall efficacy among all the models on HPRD50. Supplementary Figure S15 presents a visual comparison of the performance results on the HPRD50 dataset between BERT and GPT-based models. Supplementary Table S6 provides the actual scores of all these models and settings for the HPRD50 dataset.

Our analysis revealed interesting trends in the performance of different variations of LLMs, where we focus on autoregressive language models and masked language models in PPI prediction tasks. Specifically, BERT-based models generally showed superior performance in complex datasets like IEPA and HPRD50 compared to GPT models. On the other hand, when the datasets are less complex and more direct, as with LLL, the differences in performance between BERT and GPT models narrow, indicating that the choice of model should be tailored to the specific characteristics of the dataset at hand.

## 4 Discussion

It is worth noting that although BERT-based models demonstrate impressive performance, they require fine-tuning with supervised learning, which takes considerable time and technical expertise. In comparison, zero-shot learning models such as GPT-3.5 and GPT-4 do not require such extensive fine-tuning, making them more accessible and practical for specific use cases. This is because GPT models are much larger in terms of model parameters and pretrained on more datasets than BERT-based models. Therefore, despite being primarily designed for text generation, GPT-3.5 and GPT-4 have demonstrated remarkable ability in identifying PPIs from biomedical literature.

The major difference among our six prompt settings is that for half of them (base prompt, base prompt with protein dictionary, and base prompt with normalized protein dictionary), we used the original sentences as the input. For the other half of the settings, we used *PROTEIN* masked sentences, namely 10-fold input (identical folds used for BERT-based models), N-fold input (with no repeated sentence in the same fold), and one sentence at a time as input. The reason behind using *PROTEIN* masked sentences is to make sure there is no issue of data leakage due to using original protein names. We conducted a thorough comparison of GPT and BERT-based models with identical 10-fold settings to maintain consistency across all models, as well as explored other possible settings.

From the experimental results, we observed that BERT-based models, including BioBERT, PubMedBERT, and SciBERT, tend to show less variability in performance across the different datasets compared to GPT-based models. This could be due to the variation of BERT-based models we have used, which have a similar model architecture. Another potential reason could be that the BERT-based models have been pretrained on biomedical domain-specific corpora, which might make them more robust to variations in the protein interaction datasets. GPT-based models have competitive performance in many configurations but seem more sensitive to the prompt structure and dictionary type, possibly due to GPT’s broader language model training.

The performance also varies across datasets for all the models, which implies that each dataset has its own set of challenges. For example, the IEPA dataset has longer protein names on average, which might affect model performance if the model has limitations in processing longer sequences. This may be why the models work better on IEPA and HPRD50 in the case of *PROTEIN* masked settings compared to original sentences. However, the use of variation in the same sentence for different *PROTEIN1*-*PROTEIN2* location pairs appears to affect the performance. This could indicate the models’ sensitivity to the sentences given in the same input despite explicitly informing it to consider each sentence separately. To address this issue, the approach of processing one sentence at a time was investigated, revealing that GPT-4 notably excels with more complex datasets like HPRD50 and IEPA, suggesting its superior handling of intricate dataset characteristics.

While our study evaluated BERT- and GPT-based models, various other studies used traditional machine learning approaches for PPI identification (Miwa *et al.* 2009, Bui *et al.* 2011, Yu *et al.* 2018) and reported their performance using the same datasets used in our study. These results are also included in Supplementary Tables S4–S6, featuring comprehensive performance scores of our models. Traditional machine learning approaches have demonstrated solid performance for PPI identification. They are often lightweight and readily deployable. These methods depend heavily on handcrafted features, such as kernel-based techniques (Miwa *et al.* 2009), semantic properties (Bui *et al.* 2011), and grammatical relationship graphs (Yu *et al.* 2018), which can pose challenges regarding scalability and adaptability to new datasets or domains. Typically, best-performing traditional models require complex and time-consuming feature extraction processes that demand significant domain expertise (L’heureux *et al.* 2017, Elshawi *et al.* 2019). In contrast, LLMs offer a distinct advantage by learning directly from raw text, eliminating the need for extensive feature engineering. This capability makes LLMs more efficient and flexible for complex tasks like PPI identification in diverse biomedical datasets while ensuring comparable performance to traditional methods.

This study, although offering interesting insights, has some limitations. Initially, our investigation is limited exclusively to GPT models, specifically versions 3.5 and 4, as well as a restricted assortment of three BERT models, namely BioBERT, PubMedBERT, and SciBERT. The limited range of available LLMs may have influenced the scope of our discoveries. Furthermore, the datasets used were not large enough. The generalizability of our results to more diverse datasets may be compromised due to the limited breadth of our conclusions, which is influenced by the volume and variety of the data. Besides, using LLMs such as GPT comes with inherent challenges, notably the high computational and financial costs associated with their deployment and maintenance. This factor is particularly relevant when considering the practical implementation of these models in real-life situations.

Nevertheless, improving GPT-4 with biomedical corpora like PubMed and PMC for PPI identification is warranted. With additional information, such as a dictionary, GPT has shown decent performance and can demonstrate substantially improved performance comparable to BERT-based models, indicating the potential use of GPT for these NLP tasks. Further research is needed to explore and enhance the capabilities of GPT-based models in the biomedical domain.

Our future goal is to address the identified limitations by expanding our research to include a wider variety of models and larger, more diverse datasets. We also aim to perform domain-specific fine-tuning of GPT-based models for PPI identification. Domain-specific fine-tuning can leverage the unique characteristics of biological texts and datasets, enhancing the models’ ability to understand and predict complex PPI networks with higher accuracy and efficiency. Additionally, we intend to explore strategies for the cost-effective implementation of LLMs in practical settings, ensuring that their application is not only theoretically sound but also financially viable. Another key direction for our future research involves investigating the role of ontology in enhancing PPI literature mining. This exploration will focus on understanding how ontological frameworks can support and improve the efficiency and accuracy of extracting relevant information from biomedical texts. For example, we have previously developed the Interaction Network Ontology (INO) and have applied the INO for the mining of gene–gene or PPIs (Hur *et al.* 2015, Hur *et al.* 2017). Our study showed that the INO ontology-based literature mining enhanced the mining of the gene–gene or PPIs. Each ontology term is associated with a list of keywords supporting enhanced literature mining. Meanwhile, ontology also provides semantic relations and hierarchical structure among different interactions, which provides a basis for further interpretation and analysis of the mined interactions. While the currently reported study did not apply ontology, we plan to investigate how the ontology can be used together with existing literature mining tools to enhance our mining performance further.

Our work highlights the capabilities of GPT and BERT-based algorithms in automatically identifying PPIs from biological literature. BERT-based models like BioBERT demonstrate robust performance due to their pretraining on domain-specific corpora, resulting in high recall and F1 scores. On the other hand, GPT models, like GPT-4, demonstrate impressive zero-shot learning skills by accomplishing competitive precision and F1 scores without any fine-tuning. Despite the limitations of model scope and dataset size, the results highlight the promise of GPT models for PPI extraction. Our future research will prioritize expanding model variety, increasing dataset diversity, and fine-tuning GPT models for domain-specific tasks.

## Supplementary Material

vbae133_Supplementary_Data

## Data Availability

The source code and datasets used in this study are available at https://github.com/hurlab/PPI-GPT-BERT/.
