# Supplementary material for: Evaluating GPT and BERT models for protein–protein interaction identification in biomedical text
Source: Bioinform Adv. 2024 Sep 11;4(1):vbae133. doi: 10.1093/bioadv/vbae133 (PMC11419952; doi:10.1093/bioadv/vbae133)
Supplement: vbae133_Supplementary_Data [file vbae133_supplementary_data.docx]

**Supplementary Information for**

**Evaluating GPT and BERT models for Protein-Protein Interaction identification in biomedical text**

**Hasin Rehana et al.**

**Supplementary Figures**


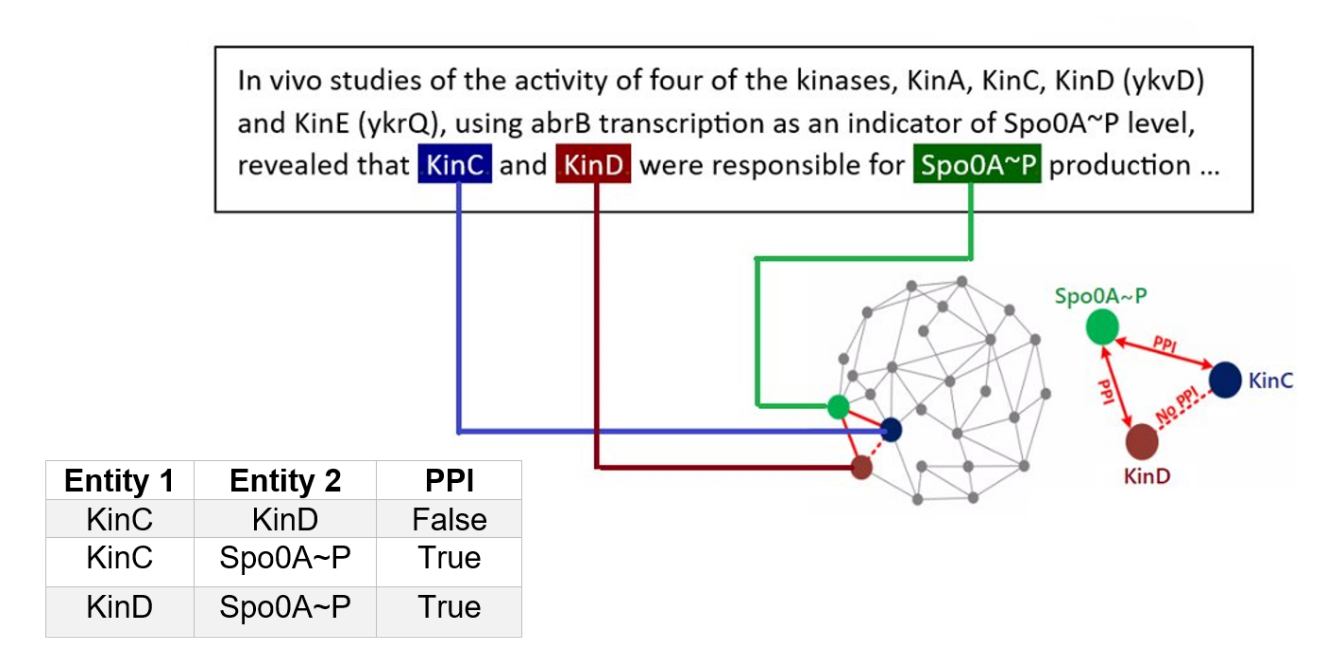


**Supplementary Fig. S1. Protein-Protein Interaction (PPI) mentioning in a biomedical text.** This figure represents a sentence segment from biomedical literature that provides information about a few proteins. The target of PPI identification is to extract protein pairs and the possible interactions between them. Here, ‘True’ means interaction, and ‘False’ indicates no interaction between the two proteins.

**
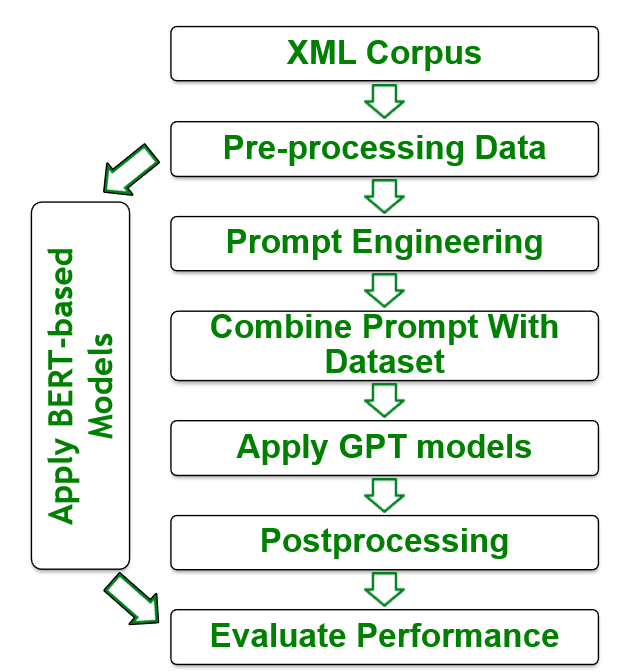
**

**Supplementary Fig. S2. Overview of methodology.**This figure outlines the step-by-step process employed in the study. The XML corpus is first pre-processed to prepare the data. Following this, prompt engineering is performed, combining the prompt with the dataset. GPT models are then applied to this combined data. Post-processing of the results is conducted before evaluating the overall performance of the models. On the other hand, BERT-based models are applied to the pre-processed data, and the performance is evaluated.


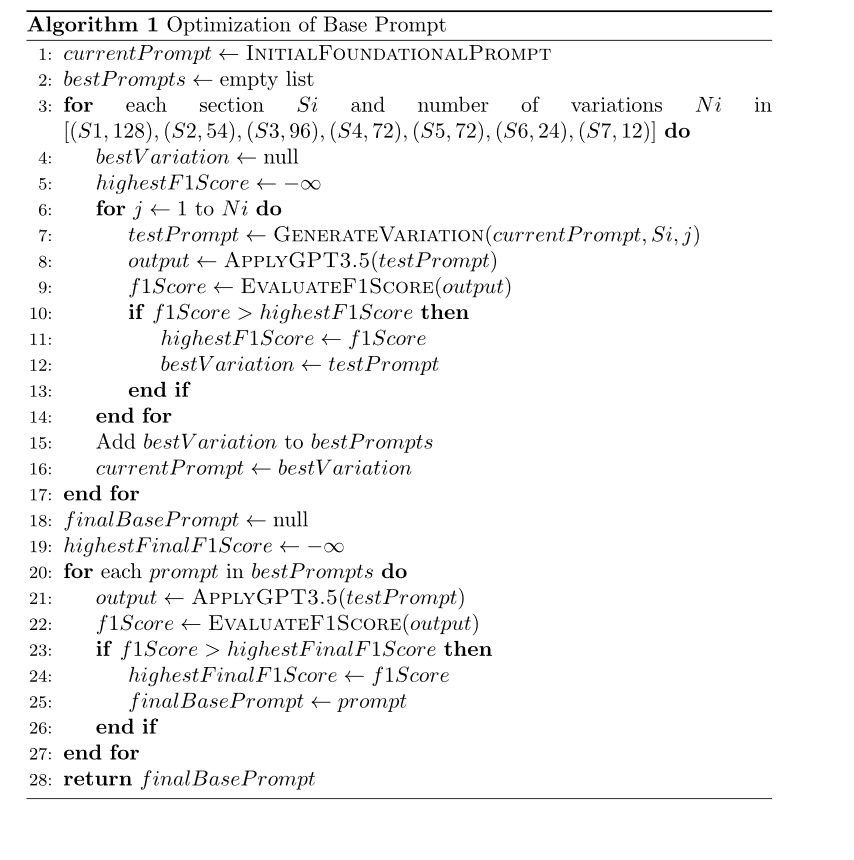


**Supplementary Fig. S3. Algorithm for prompt engineering for optimizing the best prompt.** This pseudocode illustrates the prompt engineering algorithm we used to finalize the base prompt for identifying PPI. This algorithm aims to maintain the foundational prompt constant while testing each section of the prompt separately to identify the most effective prompt variation using the F1 score as a performance metric. This evaluation was conducted sequentially for each section.


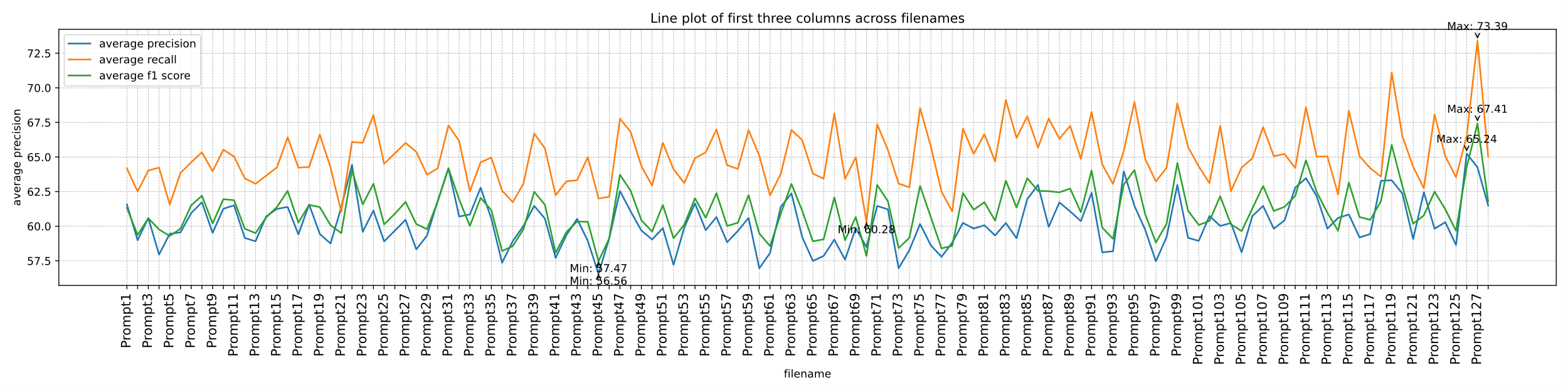


**Supplementary Fig. S4. Prompt Engineering for Base Prompt- Section 1**. This plot indicates that Prompt 127 has the best recall and F1 score for the variations of Section 1. So, Prompt 127 from Section 1 (P127_S1) is selected as the base for the prompt variation of Section 2.


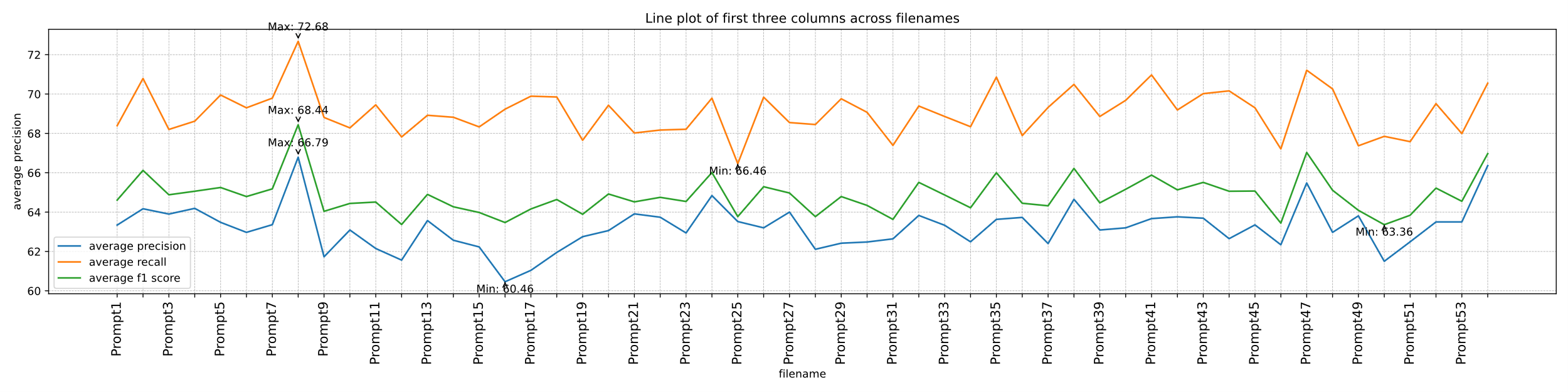


**Supplementary Fig. S5. Prompt Engineering for Base Prompt- Section 2.** This plot indicates that Prompt 8 has the best precision, recall and F1 score for the variations of Section 2. Thus, Prompt 8 from Section 2 (P8_S2) is selected as the base for the prompt variation of Section 3.


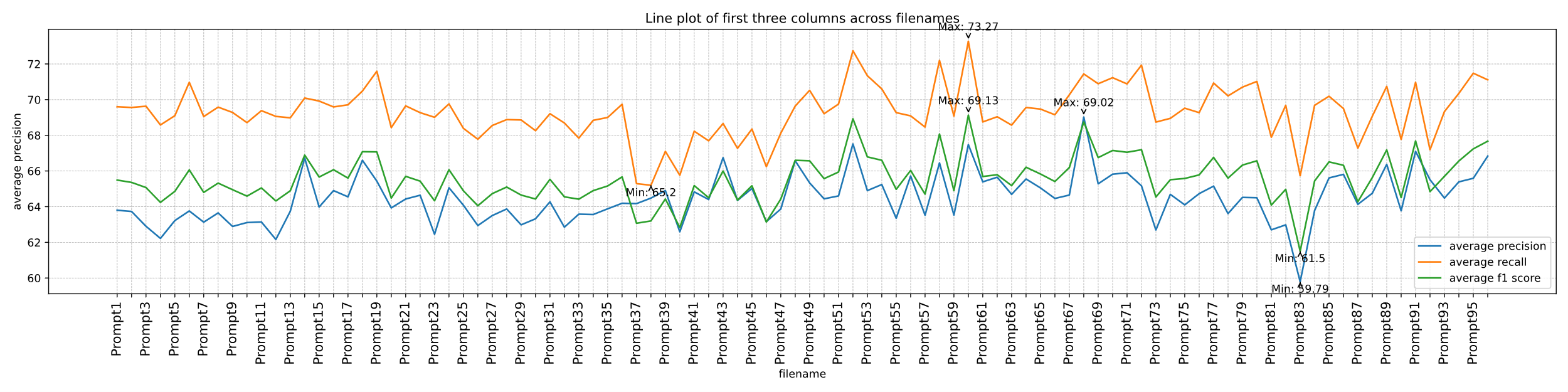


**Supplementary Fig. S6. Prompt Engineering for Base Prompt- Section 3.** This plot indicates that Prompt 60 has the best recall and F1 score for the variations of Section 3. Thus, Prompt 60 from Section 3 (P60_S3) is selected as the base for the prompt variation of Section 3.


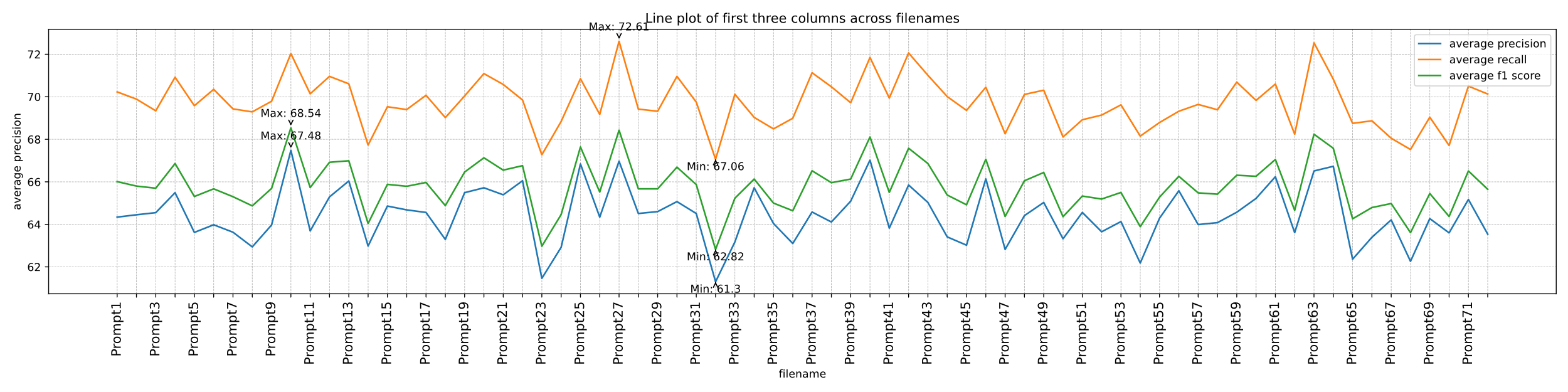


**Supplementary Fig. S7. Prompt Engineering for Base Prompt- Section 4.** This plot indicates that Prompt 10 has the best precision and F1 score for the variations of Section 4. Thus, Prompt 10 from Section 4 (P10_S4) is selected as the base for the prompt variation of Section 5.


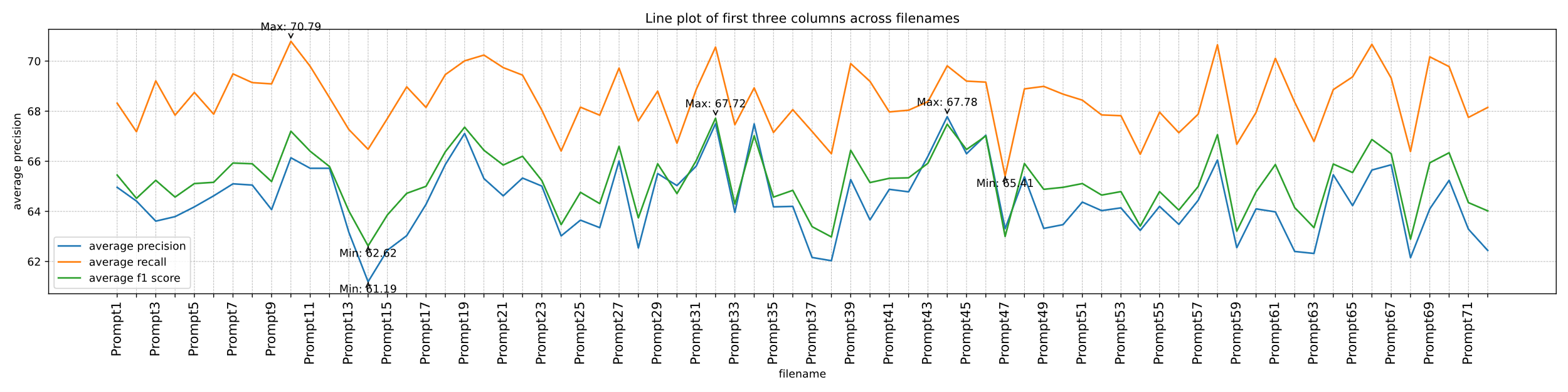


**Supplementary Fig. S8. Prompt Engineering for Base Prompt- Section 5.** This plot indicates that Prompt 32 has the best F1 score for the variations of Section 5. Thus, Prompt 32 from Section 5 (P32_S5) is selected as the base for the prompt variation of Section 6.


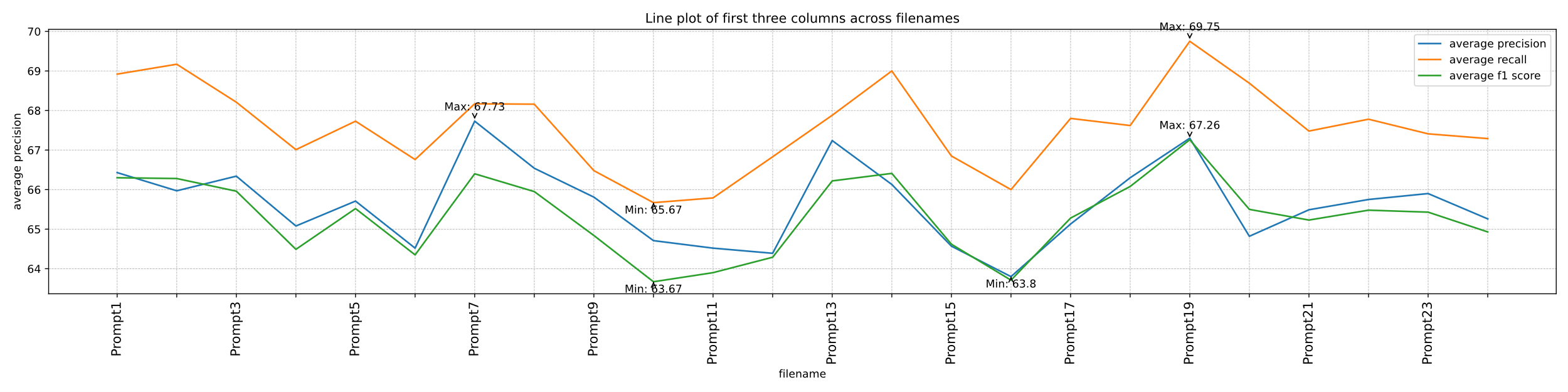


**Supplementary Fig. S9. Prompt Engineering for Base Prompt- Section 6.** This plot indicates that Prompt 19 has the best recall and F1 score for the variations of Section 6. Thus, Prompt 19 from Section 6 (P19_S6) is selected as the base for the prompt variation of Section 7.


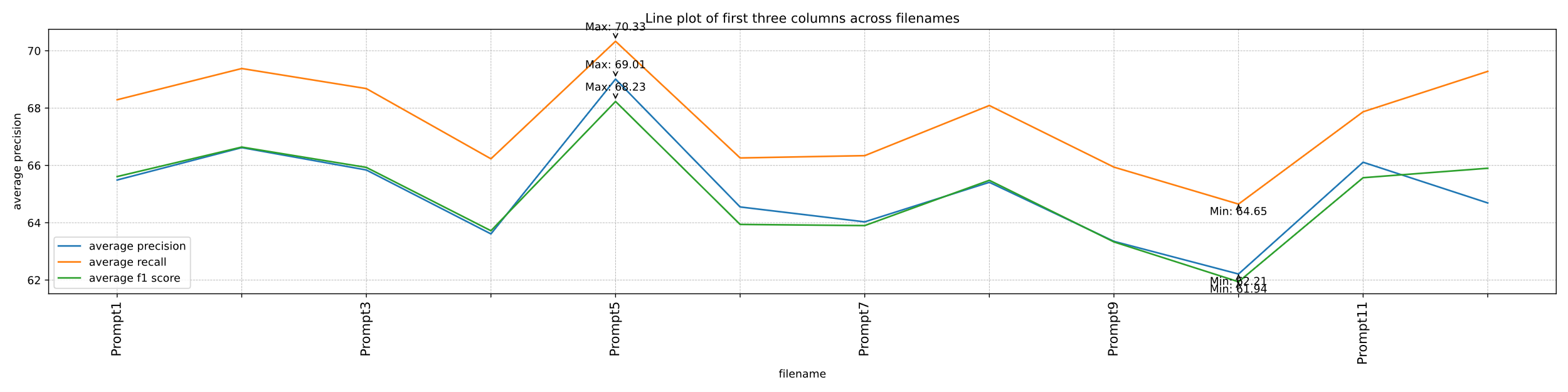


**Supplementary Fig. S10. Prompt Engineering for Base Prompt- Section 7**. This plot indicates that Prompt 5 has the best precision, recall and F1 score for the variations of Section 7. Thus, Prompt 8 from Section 2 (P8_S2) is selected as the best one from section 7.


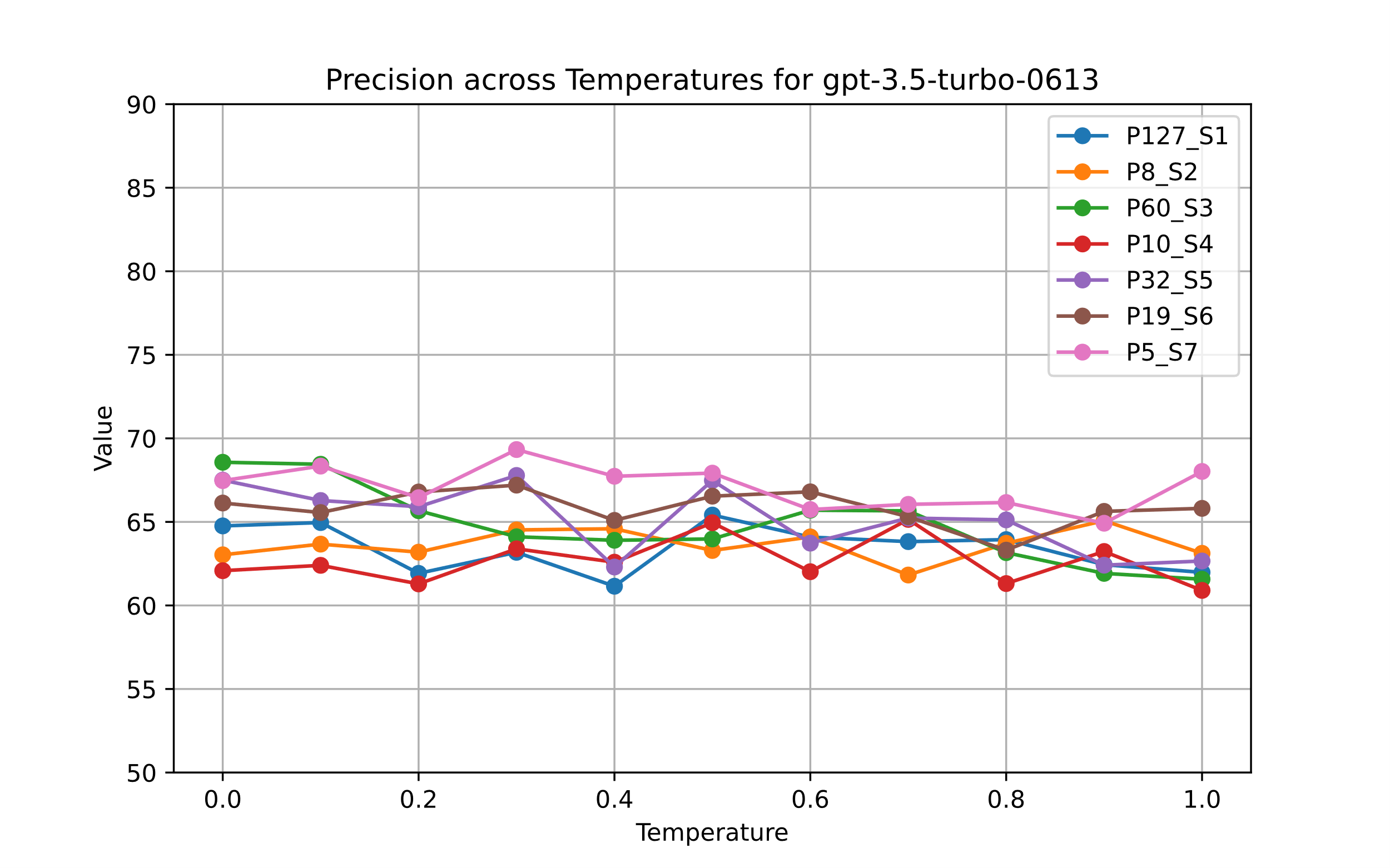


**Supplementary Fig. S11. Precision evaluation of the temperature parameter of GPT-3.5-0613 for the best prompts from each section.** This plot represents precision scores for temperature starting from 0.0 to 1.0. Here, PX_SY means Prompt number X from Section number Y. The variation in precision is very low.


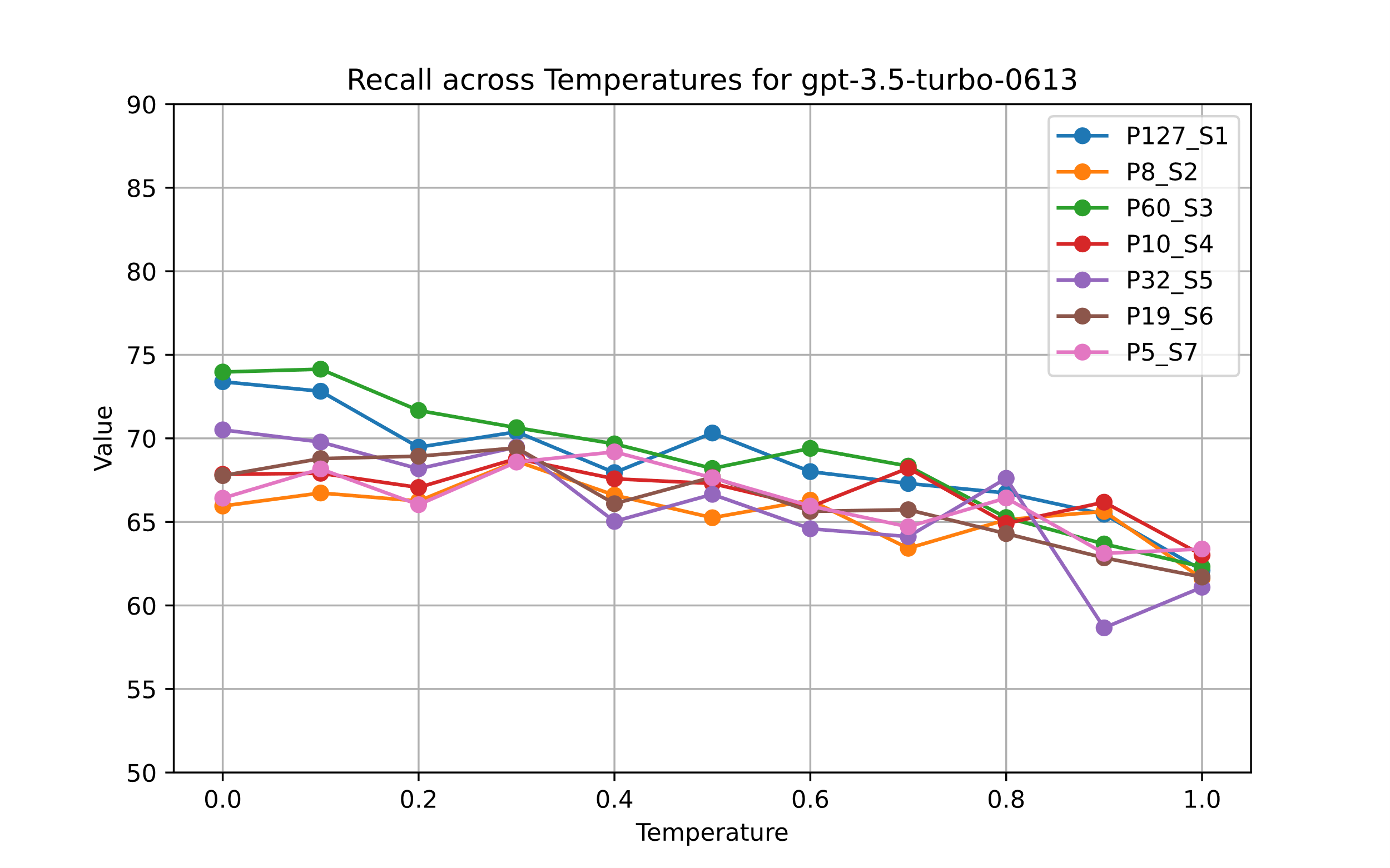


**Supplementary Fig. S12. Recall evaluation of the temperature parameter of GPT-3.5-0613 for the best prompts from each section.** This plot represents recall scores for temperature starting from 0.0 to 1.0. Here, PX_SY means prompt number X from section number Y. For almost all the best prompts from each section the recall score decreases with the increase of temperature.


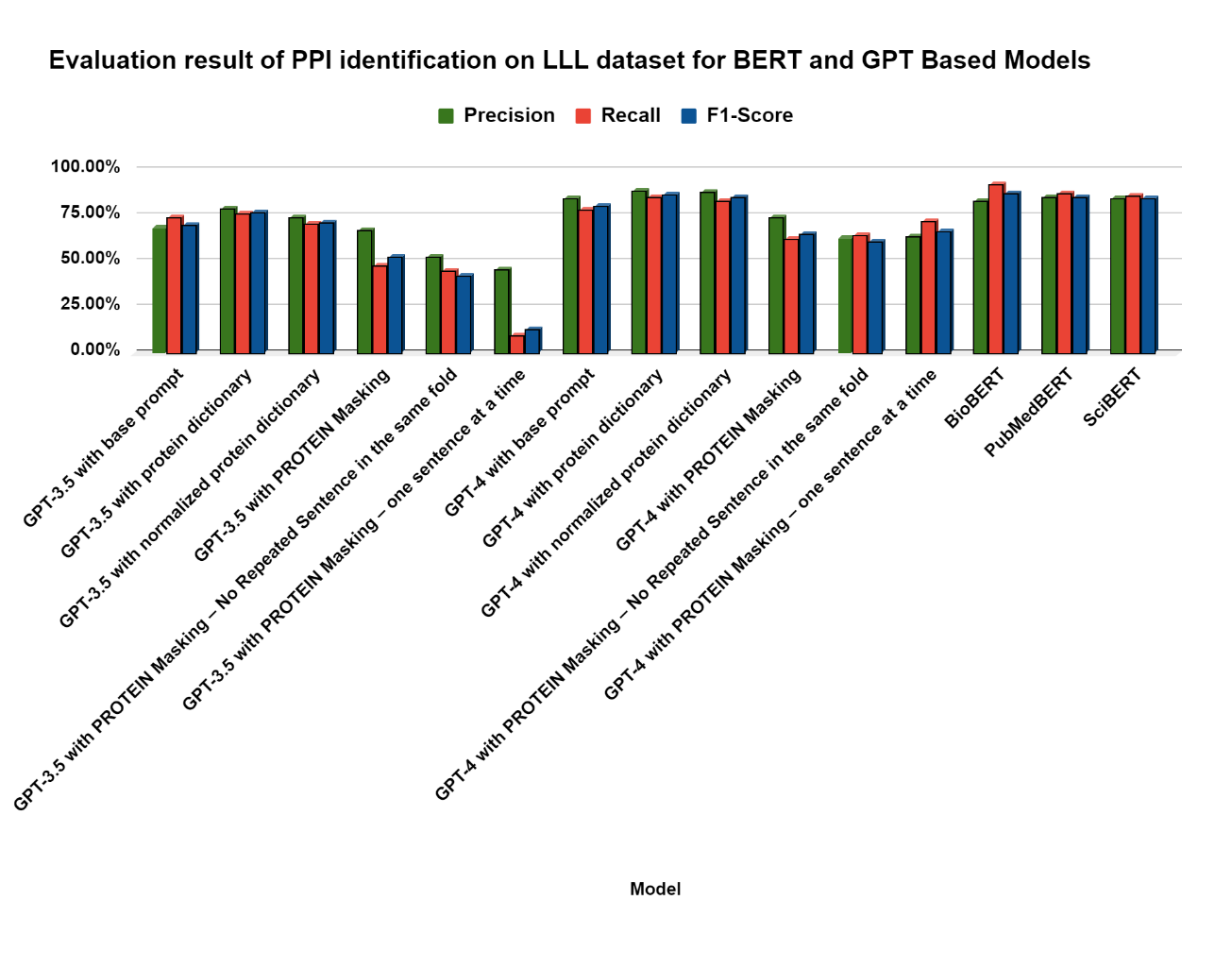


**Supplementary Fig. S13. Evaluation result of PPI identification on LLL dataset for BERT and GPT Based Models.** This bar plot presents a comparative visualization of the performance of BERT and GPT-based models in PPI identification on the LLL dataset. GPT-4 exhibits remarkable performance, especially when enhanced with a Protein dictionary. Its precision rate of 88.37% stands out, which notably exceeds that of the BERT-based models.


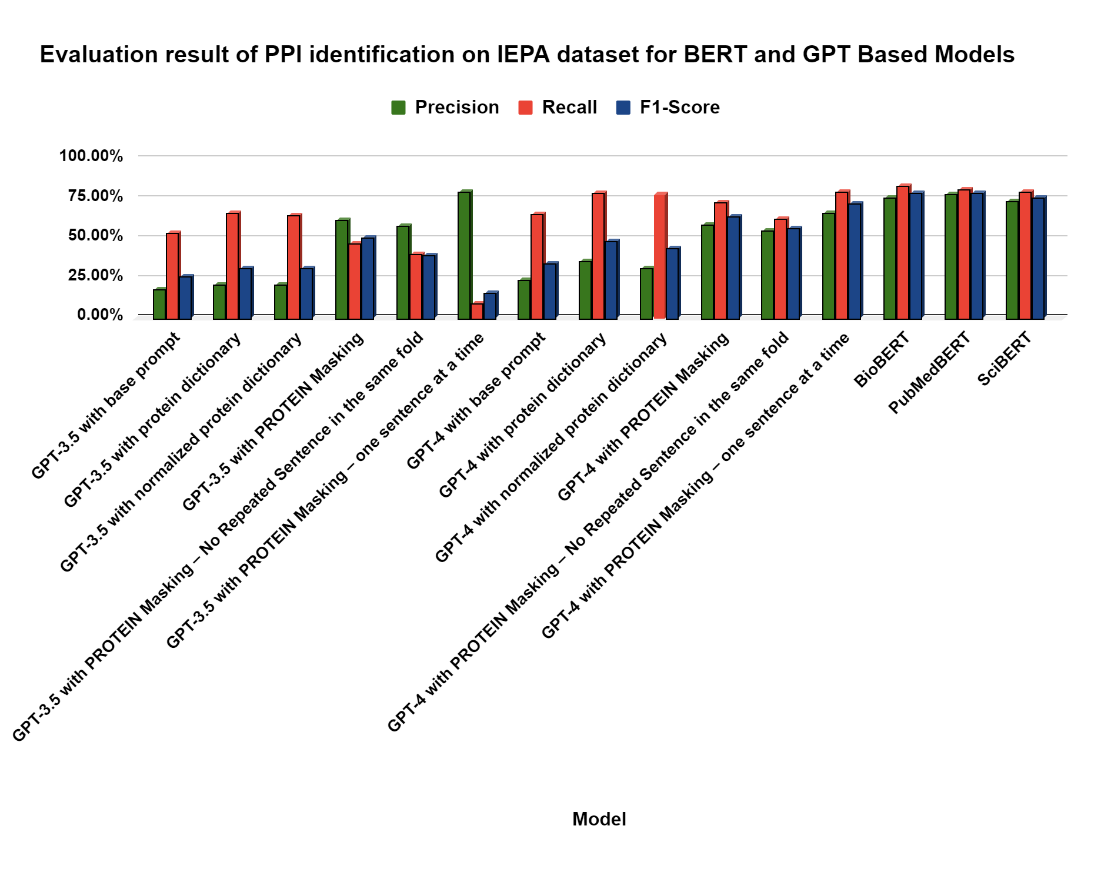
 **Supplementary Fig. S14. Evaluation result of PPI identification on IEPA dataset for BERT and GPT Based Models.** This bar plot visually compares the performance results on the HPRD50 dataset between BERT and GPT-based models. GPT-4 with PROTEIN masking (one sentence at a time) impressively achieves the best recall of 95.22%. However, both GPT-3.5 and GPT-4 lag behind BERT-based models in terms of precision and F1 score. Among all the BERT-based models, PubMedBERT achieved the highest performance metrics with a precision of 78.81%, a recall of 82.71%, and an F1 score of 79.65% for the HPRD50 dataset.


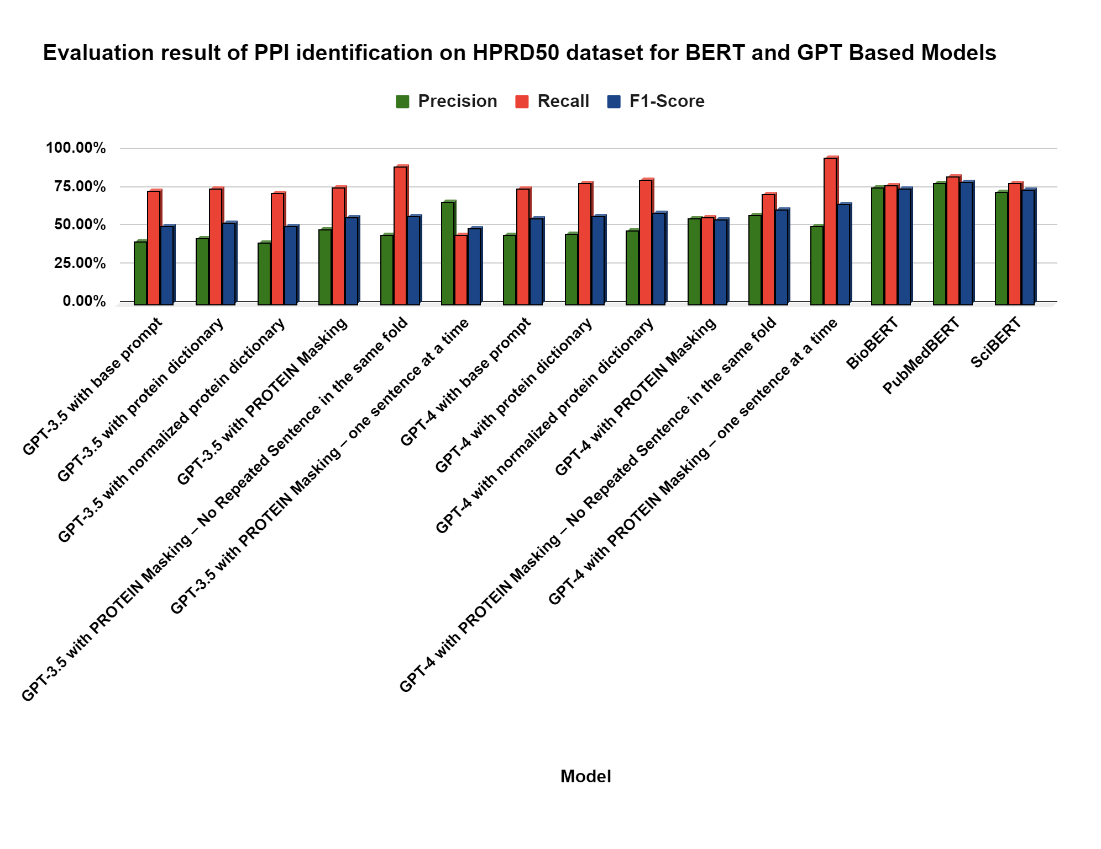


**Supplementary Fig. S15. Evaluation result of PPI identification on HPRD50 dataset for BERT and GPT Based Models.** This bar plot illustrates a comparative performance on the IEPA dataset. Among all settings of GPT-based models, GPT3.5 with *PROTEIN* masking (one sentence at a time) has the highest precision (78.95%) and GPT-4 with *PROTEIN* masking (one sentence at a time) has the highest recall (79.41%) and F1 score (71.54%). However, BERT-based models outperform GPT-based models in terms of recall and F1 score.

**Supplementary Tables**

**Supplementary Table S1. Details of Original and Normalized Dictionaries for LLL, IEPA and HPRD50 datasets.**

|  |  | Dataset | | |
| --- | --- | --- | --- | --- |
|  | Dictionary Type | LLL | IEPA | HPRD50 |
| Number of unique protein names | Original | 122 | 130 | 189 |
|  | Normalized | 103 | 100 | 182 |
| Average number of characters in protein names | Original | 4.92 | 11.8 | 8.75 |
|  | Normalized | 4.58 | 10.46 | 7.93 |
| Maximum number of characters in protein names | Original | 9 | 54 | 48 |
|  | Normalized | 8 | 50 | 44 |
| Minimum number of characters in protein names | Original | 3 | 1 | 3 |
|  | Normalized | 3 | 1 | 3 |

**Supplementary Table S2. Prompt Engineering for Base Prompt- Best Prompts from each section.**

| **Prompt** | **Prompt** |
| --- | --- |
| P127_S1 | Consider each sentence separately and infer every pair of Protein-Protein Interactions from the provided sentences.  For this task, 'Proteins' and 'Genes' are synonymous.  If a sentence contains multiple PPI pairs, list each pair on a distinct row.  Please, format your results in CSV (comma-separated values) format with the following four columns: 'Sentence ID', 'Protein 1', 'Protein 2', and 'Interaction Type'. Ensure that no columns are left blank.  Output Column Specifications:  'Sentence ID': The unique identifier for each sentence.  'Protein 1' and 'Protein 2': The entities in the sentence, representing the proteins or genes.  'Interaction Type': The type of interaction identified between the protein entities (e.g., 'binds to', 'inhibits').  If all sentences have been processed successfully, the last row should only contain the word 'Done'.  Each input line contains a 'Sentence ID' and corresponding 'Sentence' that is needed to be analyzed for finding PPI.  Here are the sentences that you need to process: |
| P8_S2 | Consider each sentence separately and infer every pair of Protein-Protein Interactions from the provided sentences.  For this task, consider Proteins and Genes as interchangeable terms.  If a sentence contains multiple PPI pairs, list each pair on a distinct row.  Please, format your results in CSV (comma-separated values) format with the following four columns: 'Sentence ID', 'Protein 1', 'Protein 2', and 'Interaction Type'. Ensure that no columns are left blank.  Output Column Specifications:  'Sentence ID': The unique identifier for each sentence.  'Protein 1' and 'Protein 2': The entities in the sentence, representing the proteins or genes.  'Interaction Type': The type of interaction identified between the protein entities (e.g., 'binds to', 'inhibits').  If all sentences have been processed successfully, the last row should only contain the word 'Done'.  Each input line contains a 'Sentence ID' and corresponding 'Sentence' that is needed to be analyzed for finding PPI.  Here are the sentences that you need to process: |
| P60_S3 | Consider each sentence separately and infer every pair of Protein-Protein Interactions from the provided sentences.  For this task, consider Proteins and Genes as interchangeable terms.  Provide each pair in a separate row whenever a sentence contains multiple Protein-Protein interaction pairs.  Please, format your results in CSV (comma-separated values) format with the following four columns: 'Sentence ID', 'Protein 1', 'Protein 2', and 'Interaction Type'. Ensure that no columns are left blank.  Output Column Specifications:  'Sentence ID': The unique identifier for each sentence.  'Protein 1' and 'Protein 2': The entities in the sentence, representing the proteins or genes.  'Interaction Type': The type of interaction identified between the protein entities (e.g., 'binds to', 'inhibits').  If all sentences have been processed successfully, the last row should only contain the word 'Done'.  Each input line contains a 'Sentence ID' and corresponding 'Sentence' that is needed to be analyzed for finding PPI.  Here are the sentences that you need to process: |
| P10_S4 | Consider each sentence separately and infer every pair of Protein-Protein Interactions from the provided sentences.  For this task, consider Proteins and Genes as interchangeable terms.  Provide each pair in a separate row whenever a sentence contains multiple Protein-Protein interaction pairs.  Please format the output in CSV with the following four columns: 'Sentence ID', 'Protein 1', 'Protein 2', and 'Interaction Type'. Ensure that no columns are left blank.  Output Column Specifications:  'Sentence ID': The unique identifier for each sentence.  'Protein 1' and 'Protein 2': The entities in the sentence, representing the proteins or genes.  'Interaction Type': The type of interaction identified between the protein entities (e.g., 'binds to', 'inhibits').  If all sentences have been processed successfully, the last row should only contain the word 'Done'.  Each input line contains a 'Sentence ID' and corresponding 'Sentence' that is needed to be analyzed for finding PPI.  Here are the sentences that you need to process: |
| P32_S5 | Consider each sentence separately and infer every pair of Protein-Protein Interactions from the provided sentences.  For this task, consider Proteins and Genes as interchangeable terms.  Provide each pair in a separate row whenever a sentence contains multiple Protein-Protein interaction pairs.  Please format the output in CSV with the following four columns: 'Sentence ID', 'Protein 1', 'Protein 2', and 'Interaction Type'. Ensure that no columns are left blank.  Output Column Specifications:  'Sentence ID': The unique ID for each sentence.  'Protein 1' and 'Protein 2': The entity pairs in the sentence representing the proteins or genes with potential PPI.  'Interaction Type': The type of interaction identified between the protein pairs (e.g., 'binds to', 'inhibits').  If all sentences have been processed successfully, the last row should only contain the word 'Done'.  Each input line contains a 'Sentence ID' and corresponding 'Sentence' that is needed to be analyzed for finding PPI.  Here are the sentences that you need to process: |
| P19_S6 | Consider each sentence separately and infer every pair of Protein-Protein Interactions from the provided sentences.  For this task, consider Proteins and Genes as interchangeable terms.  Provide each pair in a separate row whenever a sentence contains multiple Protein-Protein interaction pairs.  Please format the output in CSV with the following four columns: 'Sentence ID', 'Protein 1', 'Protein 2', and 'Interaction Type'. Ensure that no columns are left blank.  Output Column Specifications:  The last output row should exclusively contain the word 'Done' to indicate that all the sentences have been processed successfully.  'Protein 1' and 'Protein 2': The entity pairs in the sentence representing the proteins or genes with potential PPI.  'Interaction Type': The type of interaction identified between the protein pairs (e.g., 'binds to', 'inhibits').  If all sentences have been processed successfully, the last row should only contain the word 'Done'.  Each input line contains a 'Sentence ID' and corresponding 'Sentence' that is needed to be analyzed for finding PPI.  Here are the sentences that you need to process: |
| P5_S7 | Consider each sentence separately and infer every pair of Protein-Protein Interactions from the provided sentences.  For this task, consider Proteins and Genes as interchangeable terms.  Provide each pair in a separate row whenever a sentence contains multiple Protein-Protein interaction pairs.  Please format the output in CSV with the following four columns: 'Sentence ID', 'Protein 1', 'Protein 2', and 'Interaction Type'. Ensure that no columns are left blank.  Output Column Specifications:  The last output row should exclusively contain the word 'Done' to indicate that all the sentences have been processed successfully.  'Protein 1' and 'Protein 2': The entity pairs in the sentence representing the proteins or genes with potential PPI.  'Interaction Type': The type of interaction identified between the protein pairs (e.g., 'binds to', 'inhibits').  If all sentences have been processed successfully, the last row should only contain the word 'Done'.  Each input line contains a 'Sentence ID' and its corresponding 'Sentence' for PPI analysis.  Here are the sentences that you need to process: |

# Prompt PX_SY means Prompt number X from Section number Y

**Supplementary Table S3. Final Prompts for each Prompt Type.**

| **Prompt Type** | **Prompt** |
| --- | --- |
| **Base**  **(10 fold)** | Consider each sentence separately and infer every pair of Protein-Protein Interactions from the provided sentences.  For this task, consider Proteins and Genes as interchangeable terms.  Provide each pair in a separate row whenever a sentence contains multiple Protein-Protein interaction pairs.  Please, format your results in CSV (comma-separated values) format with the following four columns: 'Sentence ID', 'Protein 1', 'Protein 2', and 'Interaction Type'. Ensure that no columns are left blank.  Output Column Specifications:  'Sentence ID': The unique identifier for each sentence.  'Protein 1' and 'Protein 2': The entities in the sentence, representing the proteins or genes.  'Interaction Type': The type of interaction identified between the protein entities (e.g., 'binds to', 'inhibits').  If all sentences have been processed successfully, the last row should only contain the word 'Done'.  Each input line contains a 'Sentence ID' and corresponding 'Sentence' that is needed to be analyzed for finding PPI.  Here are the sentences that you need to process: |
| **With Protein Dictionary (10 fold)** | Please, format your results in CSV (comma-separated values) format with the following four columns: 'Sentence ID', 'Protein 1', 'Protein 2', and 'Interaction Type'. Ensure that no columns are left blank.  Output Column Specifications:  'Sentence ID': The unique identifier for each sentence.  'Protein 1' and 'Protein 2': The entities in the sentence, representing the proteins or genes.  'Interaction Type': The type of interaction identified between the protein entities (e.g., 'binds to', 'inhibits').  If all sentences have been processed successfully, the last row should only contain the word 'Done'.  Each input line contains a 'Sentence ID' and corresponding 'Sentence' that is needed to be analyzed for finding PPI.  Here are the protein names for your reference : [['KinC' 'KinD' 'sigma(A)' 'Spo0A' 'SigE' 'SigK' 'GerE' 'sigma(F)' 'sigma(G)' 'SpoIIE' 'FtsZ' 'sigma(H)' 'sigma(K)' 'gerE' 'EsigmaF' 'sigmaB' 'sigmaF' 'SpoIIAB' 'SpoIIAA' 'SigL' 'RocR' 'sigma(54)' 'E sigma E' 'YfhP' 'SpoIIAA-P' 'sigmaK' 'sigmaG' 'ComK' 'FlgM' 'sigma X' 'sigma B' 'sigma(B)' 'sigmaD' 'SpoIIID' 'sigmaW' 'PhoP~P' 'AraR' 'sigmaH' 'yvyD' 'ClpX' 'Spo0' 'RbsW' 'DnaK' 'sigmaE' 'sigma W' 'sigmaA' 'sigma(X)' 'CtsR' 'Spo0A~P' 'spoIIG' 'ydhD' 'ykuD' 'ykvP' 'ywhE' 'spo0A' 'spoVG' 'rsfA' 'cwlH' 'KatX' 'katX' 'rocG' 'yfhS' 'yfhQ' 'yfhR' 'sspE' 'yfhP' 'bmrUR' 'ydaP' 'ydaE' 'ydaG' 'yfkM' 'sigma F' 'cot' 'sigK' 'cotD' 'sspG' 'sspJ' 'hag' 'comF' 'flgM' 'ykzA' 'CsbB' 'nadE' 'YtxH' 'YvyD' 'bkd' 'degR' 'cotC' 'cotX' 'cotB' 'sigW' 'tagA' 'tagD' 'tuaA' 'araE' 'sigmaL' 'spo0H' 'sigma G' 'sigma 28' 'sigma 32' 'spoIVA' 'PBP4*' 'RacX' 'YteI' 'YuaG' 'YknXYZ' 'YdjP' 'YfhM' 'phrC' 'sigE' 'ald' 'kdgR' 'sigX' 'ypuN' 'clpC' 'ftsY' 'gsiB' 'sigB' 'sspH' 'sspL' 'sspN' 'tlp']]  Here are the sentences that you need to process: |
| **With Normalized Protein Dictionary (10 fold)** | Please, format your results in CSV (comma-separated values) format with the following four columns: 'Sentence ID', 'Protein 1', 'Protein 2', and 'Interaction Type'. Ensure that no columns are left blank.  Output Column Specifications:  'Sentence ID': The unique identifier for each sentence.  'Protein 1' and 'Protein 2': The entities in the sentence, representing the proteins or genes.  'Interaction Type': The type of interaction identified between the protein entities (e.g., 'binds to', 'inhibits').  If all sentences have been processed successfully, the last row should only contain the word 'Done'.  Each input line contains a 'Sentence ID' and corresponding 'Sentence' that is needed to be analyzed for finding PPI.  Here are the normalized protein names for your reference : [['kinc' 'kind' 'sigmaa' 'spo0a' 'sige' 'sigk' 'gere' 'sigmaf' 'sigmag' 'spoiie' 'ftsz' 'sigmah' 'sigmak' 'esigmaf' 'sigmab' 'spoiiab' 'spoiiaa' 'sigl' 'rocr' 'sigma54' 'esigmae' 'yfhp' 'spoiiaa-p' 'comk' 'flgm' 'sigmax' 'sigmad' 'spoiiid' 'sigmaw' 'phop~p' 'arar' 'yvyd' 'clpx' 'spo0' 'rbsw' 'dnak' 'sigmae' 'ctsr' 'spo0a~p' 'spoiig' 'ydhd' 'ykud' 'ykvp' 'ywhe' 'spovg' 'rsfa' 'cwlh' 'katx' 'rocg' 'yfhs' 'yfhq' 'yfhr' 'sspe' 'bmrur' 'ydap' 'ydae' 'ydag' 'yfkm' 'cot' 'cotd' 'sspg' 'sspj' 'hag' 'comf' 'ykza' 'csbb' 'nade' 'ytxh' 'bkd' 'degr' 'cotc' 'cotx' 'cotb' 'sigw' 'taga' 'tagd' 'tuaa' 'arae' 'sigmal' 'spo0h' 'sigma28' 'sigma32' 'spoiva' 'pbp4*' 'racx' 'ytei' 'yuag' 'yknxyz' 'ydjp' 'yfhm' 'phrc' 'ald' 'kdgr' 'sigx' 'ypun' 'clpc' 'ftsy' 'gsib' 'sigb' 'ssph' 'sspl' 'sspn' 'tlp']]  Here are the sentences that you need to process: |
| **With PROTEIN masking (10 fold)** | Consider each sentence separately and infer Protein-Protein Interaction for the protein entity pairs PROTEIN1-PROTEIN2 from the provided sentences. Do not consider any other PROTEIN pairs in the sentence. In each sentence, original protein or gene names have been substituted with 'PROTEIN1', 'PROTEIN', and 'PROTEIN' placeholders. The placeholders used may represent a variety of proteins or genes, differing with each sentence.  Please, format your results in CSV (comma-separated values) with only two columns: 'Sentence ID' and 'PPI'. Do not include the original sentences or any explanation in the output.  Output Column Specifications:  'Sentence ID': The unique identifier for each sentence.  'PPI': Record your findings as 'TRUE' if there is a demonstrable interaction between PROTEIN1 and PROTEIN2, and 'FALSE' if there is none.  If all sentences have been processed successfully, the last row should only contain the word 'Done'.  Each input line contains a 'Sentence ID' and corresponding 'Sentence' that is needed to be analyzed for finding PPI.  Here are the sentences that you need to process: |
| **With PROTEIN masking (Nfold)** | Consider each sentence separately and infer Protein-Protein Interaction for the protein entity pairs PROTEIN1-PROTEIN2 from the provided sentences. Do not consider any other PROTEIN pairs in the sentence. In each sentence, original protein or gene names have been substituted with 'PROTEIN1', 'PROTEIN', and 'PROTEIN' placeholders. The placeholders used may represent a variety of proteins or genes, differing with each sentence.  Please, format your results in CSV (comma-separated values) with only two columns: 'Sentence ID' and 'PPI'. Do not include the original sentences or any explanation in the output.  Output Column Specifications:  'Sentence ID': The unique identifier for each sentence.  'PPI': Record your findings as 'TRUE' if there is a demonstrable interaction between PROTEIN1 and PROTEIN2, and 'FALSE' if there is none.  If all sentences have been processed successfully, the last row should only contain the word 'Done'.  Each input line contains a 'Sentence ID' and corresponding 'Sentence' that is needed to be analyzed for finding PPI.  Here are the sentences that you need to process: |
| **With PROTEIN masking (One Sentence at a time)** | Infer Protein-Protein Interaction for the protein entity pairs PROTEIN1-PROTEIN2 from the provided sentence. Do not consider any other PROTEIN pairs in the sentence. Original protein or gene names have been substituted with 'PROTEIN1', 'PROTEIN', and 'PROTEIN' placeholders.  Output Specification: Record your findings as 'TRUE' if there is a demonstrable interaction between PROTEIN1 and PROTEIN2, and 'FALSE' if there is none.  Here is the sentence that you need to process: |

# 10 fold - Sentences in each fold here are the same as BERT, Nfold – The same sentence with different positional PROTEIN masking is not present in the same fold.

**Supplementary Table S4:** Performance comparison of LLM models with traditional machine learning approaches on the LLL dataset.

| Type | Model | Precision | Recall | F1-Score |
| --- | --- | --- | --- | --- |
| ***Autoregressive Language Models*** | GPT-3.5 with base prompt | 68.57% | 73.97% | 70.05% |
|  | GPT-3.5 with protein dictionary | 79.06% | 75.95% | 76.72% |
|  | GPT-3.5 with normalized protein dictionary | 74.20% | 70.55% | 71.39% |
|  | GPT-3.5 with *PROTEIN* Masking | 66.90% | 47.52% | 52.68% |
|  | GPT-3.5 with *PROTEIN* Masking – No Repeated Sentence in the same fold | 52.28% | 44.85% | 42.25% |
|  | GPT-3.5 with *PROTEIN* Masking – one sentence at a time | 45.16% | 9.53% | 12.63% |
|  | GPT-4 with base prompt | 84.38% | 78.43% | 80.06% |
|  | GPT-4 with protein dictionary | **88.37%** | **85.14%** | **86.49%** |
|  | GPT-4 with normalized protein dictionary | 87.97% | 83.25% | 85.21% |
|  | GPT-4 with *PROTEIN* Masking | 73.99% | 62.48% | 64.72% |
|  | GPT-4 with *PROTEIN* Masking – No Repeated Sentence in the same fold | 62.58% | 64.07% | 60.74% |
|  | GPT-4 with *PROTEIN* Masking – one sentence at a time | 63.68% | 71.71% | 66.29% |
| ***Masked Language Models*** | BioBERT | 82.82% | **91.95%** | **86.84%** |
|  | PubMedBERT | **85.25%** | 87.35% | 85.42% |
|  | SciBERT | 84.54% | 86.07% | 84.66% |
| *Traditional Machine Learning Models* | Kernel-based machine learning, support vector machine (SVM) (Miwa et al., 2009) | 77.60% | 86.00% | 80.10% |
|  | Semantic properties, SVM (Bui et al., 2011) | **84.10%** | 84.10% | **84.10%** |
|  | Grammatical relationship graph for triplets, shortest path algorithm (Yu et al., 2018) | 83.60% | **91.20%** | 77.10% |

**Supplementary Table S5:** Performance comparison of LLM models with traditional machine learning approaches on the IEPA dataset.

| Type | Model | Precision | Recall | F1-Score |
| --- | --- | --- | --- | --- |
| ***Autoregressive Language Models*** | GPT-3.5 with base prompt | 17.58% | 53.35% | 25.53% |
|  | GPT-3.5 with protein dictionary | 20.69% | 65.92% | 31.25% |
|  | GPT-3.5 with normalized protein dictionary | 20.45% | 64.7% | 30.79% |
|  | GPT-3.5 with PROTEIN Masking | 61.55% | 46.28% | 50.62% |
|  | GPT-3.5 with PROTEIN Masking – No Repeated Sentence in the same fold | 57.74% | 39.97% | 39.34% |
|  | GPT-3.5 with PROTEIN Masking – one sentence at a time | **78.95%** | 8.69% | 15.32% |
|  | GPT-4 with base prompt | 23.66% | 64.94% | 34.19% |
|  | GPT-4 with protein dictionary | 35.36% | 78.78% | 48.37% |
|  | GPT-4 with normalized protein dictionary | 30.95% | 77.70% | 43.80% |
|  | GPT-4 with PROTEIN Masking | 58.61% | 72.71% | 64.00% |
|  | GPT-4 with PROTEIN Masking – No Repeated Sentence in the same fold | 55.06% | 62.03% | 55.89% |
|  | GPT-4 with PROTEIN Masking – one sentence at a time | 65.71% | **79.41%** | **71.54%** |
| ***Masked Language Models*** | BioBERT | 75.80% | **83.21%** | **78.81%** |
|  | PubMedBERT | **77.70%** | 81.05% | 78.49% |
|  | SciBERT | 73.29% | 79.32% | 75.53% |
| *Traditional Machine Learning Models* | Kernel-based machine learning, support vector machine (SVM) (Miwa et al., 2009) | 67.50% | 78.60% | 71.70% |
|  | Semantic properties, SVM (Bui et al., 2011) | 67.40% | 83.90% | **74.70%** |
|  | Grammatical relationship graph for triplets, shortest path algorithm (Yu et al., 2018) | **74.90%** | **91.00%** | 63.60% |

**Supplementary Table S6:** Performance comparison of LLM models with traditional machine learning approaches on the HPRD50 dataset.

| Type | Model | Precision | Recall | F1-Score |
| --- | --- | --- | --- | --- |
| ***Autoregressive Language Models*** | GPT-3.5 with base prompt | 40.62% | 73.89% | 50.96% |
|  | GPT-3.5 with protein dictionary | 42.99% | 74.80% | 52.90% |
|  | GPT-3.5 with normalized protein dictionary | 40.06% | 72.05% | 50.48% |
|  | GPT-3.5 with PROTEIN Masking | 48.13% | 76.21% | 56.33% |
|  | GPT-3.5 with PROTEIN Masking – No Repeated Sentence in the same fold | 44.70% | 89.29% | 57.26% |
|  | GPT-3.5 with PROTEIN Masking – one sentence at a time | **66.40%** | 44.72% | 49.09% |
|  | GPT-4 with base prompt | 44.83% | 75.16% | 55.33% |
|  | GPT-4 with protein dictionary | 45.90% | 78.90% | 57.24% |
|  | GPT-4 with normalized protein dictionary | 47.76% | 80.64% | 58.96% |
|  | GPT-4 with PROTEIN Masking | 55.58% | 56.24% | 54.62% |
|  | GPT-4 with PROTEIN Masking – No Repeated Sentence in the same fold | 58.06% | 71.35% | 61.24% |
|  | GPT-4 with PROTEIN Masking – one sentence at a time | 50.36% | **95.22%** | **65.00%** |
| ***Masked Language Models*** | BioBERT | 75.79% | 77.63% | 74.95% |
|  | PubMedBERT | **78.81%** | **82.71%** | **79.65%** |
|  | SciBERT | 73.03% | 79.02% | 74.67% |
| *Traditional Machine Learning Models* | Kernel-based machine learning, support vector machine (SVM) (Miwa et al., 2009) | 68.50% | 76.10% | 70.90% |
|  | Semantic properties, SVM (Bui et al., 2011) | **70.20%** | 77.90% | **73.80%** |
|  | Grammatical relationship graph for triplets, shortest path algorithm (Yu et al., 2018) | 64.00% | **86.50%** | 50.80% |

**References**

BUI, Q.-C., KATRENKO, S. & SLOOT, P. M. 2011. A hybrid approach to extract protein–protein interactions. *Bioinformatics,* 27**,** 259-265.

MIWA, M., SÆTRE, R., MIYAO, Y. & TSUJII, J. I. 2009. Protein–protein interaction extraction by leveraging multiple kernels and parsers. *International journal of medical informatics,* 78**,** e39-e46.

YU, K., LUNG, P.-Y., ZHAO, T., ZHAO, P., TSENG, Y.-Y. & ZHANG, J. 2018. Automatic extraction of protein-protein interactions using grammatical relationship graph. *BMC medical informatics and decision making,* 18**,** 35-43.
